# European Stroke Organisation (ESO) standard operating procedure for white papers (expert consensus based clinical guidance)

**DOI:** 10.1177/23969873251316430

**Published:** 2025-02-04

**Authors:** Diana Aguiar de Sousa, Annaelle Zietz, Marialuisa Zedde, Aristeidis H Katsanos, Linxin Li, Joan Marti-Fabregas, Christian H Nolte, Anna Podlasek, Sven Poli, Jan Purrucker, Melinda B Roaldsen, Peter D Schellinger, Daniel Strbian, Georgios Tsivgoulis, Sofia Tsokani, Areti Angeliki Veroniki, Terence J Quinn

**Affiliations:** 1 Lisbon Central University Hospital – ULS São José, Stroke Center, Lisbon, Portugal; 2 Gulbenkian Institute for Molecular Medicine and Faculdade de Medicina, Universidade de Lisboa, Lisbon, Portugal; 3 Department of Neurology and Stroke Centre, University Hospital Basel and University of Basel, Basel, Switzerland; 4 Neurology Unit, Stroke Unit, Azienda Unità Sanitaria Locale-IRCCS di Reggio Emilia, Reggio Emilia, Italy; 5 Division of Neurology, McMaster University & Population Health Research Institute, Hamilton, ON, Canada; 6 Wolfson Centre for Prevention of Stroke and Dementia, Nuffield Department of Clinical Neurosciences, University of Oxford, Oxford, UK; 7 John Radcliffe Hospital, Oxford, UK; 8 Department of Neurology (Stroke Unit), Hospital de la Santa Creu i Sant Pau, Barcelona, Spain; 9 Department of Neurology with Experimental Neurology, Charité, Universitätsmedizin-Berlin, Center for Stroke Research Berlin (CSB) and Berlin Institute of Health (BIH), Germany; 10 Tayside Innovation MedTech Ecosystem (TIME), University of Dundee, Dundee, Scotland, UK; 11 Department of Neurology & Stroke and Hertie Institute for Clinical Brain Research, University of Tübingen, Tübingen, Germany; 12 Heidelberg University Hospital, Heidelberg, Germany; 13 Clinical Research Department, University Hospital of North Norway, Tromsø, Norway; 14 Department of Clinical Medicine, UiT the Arctic University of Norway, Tromsø, Norway; 15 Deptartment of Neurology and Neurogeriatrics, John Wesling Medical Center Minden, UK RUB, Germany; 16 Department of Neurology, Helsinki University Central Hospital HUCH, Helsinki, Finland; 17 Second Department of Neurology, Medical School, National & Kapodistrian University of Athens, ‘Attikon’ University Hospital, Athens, Greece; 18 Laboratory of Hygiene, Social & Preventive Medicine and Medical Statistics, School of Medicine, Aristotle University of Thessaloniki, Greece; 19 Knowledge Translation Program, Li Ka Shing Knowledge Institute, StMichael’s Hospital, Toronto, ON, Canada; 20 Institute for Health Policy, Management, and Evaluation, University of Toronto, Toronto, ON, Canada; 21 School of Cardiovascular and Medical Sciences, University of Glasgow, Glasgow, UK

**Keywords:** Consensus, Delphi, evidence, guideline

## Abstract

Promoting the highest quality, evidence-based research across Europe is a priority of the European Stroke Organisation (ESO). The ESO Guideline Board communicate and promote evidence-based recommendations for clinical practice through their Guidelines. However, there are many aspects of stroke care where robust scientific evidence may be unavailable or difficult to obtain. Thus, there is a need for practical, consensus guidance, produced following robust, consistent, and transparent methods, that is suitable for high-priority clinical scenarios where evidence is currently lacking. The ESO Guideline Board developed methods for producing practical clinical guidance based on expert consensus in response to this need. These ESO’ White Papers’ are intended to complement standard ESO Guidelines. Here, we outline the ESO White Papers’ standard operating procedure (SOP). We will describe the motivation for creating White Papers, the preferred composition of writing groups and expert consensus panellists, the methods for achieving consensus, and how results will be communicated. To ensure that all voting members have an equal voice, our methods are based upon the Delphi process of repeated rounds of anonymous voting, feedback and review. We hope that the White Papers will add further value to the clinical practice guidance that is offered by ESO. We look forward to receiving suggestions for White Paper topics from the stroke community.

## Background and context – the need for White Paper consensus materials

Promoting the highest quality, evidence-based research across Europe is a priority of the European Stroke Organisation (ESO). The ESO has used clinical practice guidelines as a vehicle to raise standards and ensure consistency in practice. The ESO Guideline Board develops various products, all intended to support clinical decision-making in stroke care: clinical practice guidelines,^1^ expedited recommendations,^2^ and collaborative guidelines with other professional societies (joint guidelines or endorsements).^3^ All these materials are predicated on the availability of robust research to inform an evidence-based recommendation.

There are many aspects of stroke care where robust scientific evidence may be unavailable or may be difficult to obtain. Examples include but are not limited to, novel therapeutic areas where the published evidence is not yet mature enough to support a recommendation, rare conditions where adequately powered studies are improbable, or clinical questions in populations who have traditionally been excluded from randomised trials.^4^ In these situations, the clinical community require guidance, but the standard clinical practice guideline format is unsuitable.

Expert consensus can be useful in situations where robust evidence is lacking or limited, opinions are divergent, or collective judgement could aid in an area of equipoise. A rigorous and transparent consensus methodology ensures that the knowledge and experience of multidisciplinary thought leaders can be utilised to produce practical guidance in high-value clinical topic areas. Best practice methods in developing and reporting consensus guidance have recently been made available and will help societies ensure that expert consensus-based outputs are standardised and robust.^5^

Recognising the potential for consensus guidance, ESO has responded in two ways. ESO has already produced expert reviews under the collective title of White Papers.^6,7^ These reviews did not follow a standard approach, resulting in inconsistency in the methods used and in the format of guidance produced. Feedback to the ESO Guideline Board has been that papers offering practical clinical guidance are welcome, and indeed, the previous ‘White Papers’ have been highly cited. However, the lack of standardisation and pre-defined methods is a concern.

In parallel, traditional ESO guidelines allow for an Expert Consensus Statement. The statement is formulated through discussion and then formalised by voting amongst the panel members (module working group – MWG). These statements have been a useful addition to ESO Guidelines, but they intend to complement formal evidence-based recommendations rather than be the sole guidance offered. There are many examples of practice areas where the lack of evidence would mandate that no evidence-based recommendations could be formulated. The current ESO Guideline format and Standardised Operating Procedure (SOP) are considered unsuitable for such an offering.^8,9^

Therefore, there is an important gap in the extent of recommendations the ESO can offer clinicians. To address this need, we propose a new approach that offers practical, consensus guidance in an area where evidence is lacking and that is produced following robust, consistent, and transparent methods. This manuscript outlines the SOP to produce consistent ESO White Papers as Expert Consensus-Based Clinical Guidance, thereby filling a crucial void in clinical decision-making resources.

## Methods for producing White Papers

ESO Guidelines already benefit from an established and effective SOP.^9^ Where possible, the methods for producing a White Paper will follow the process outlined in the generic SOP. However, there will be aspects of method and reporting which necessarily deviate from the SOP. These will be highlighted in this document.

In developing our approach to consensus-based materials, we reviewed relevant literature. We collated best practices in clinical consensus building^10,11^ and reviewed consensus documents from other professional societies and guideline producers to create a format that suits the needs of the ESO community. There are various terminologies for clinical guidance from professional societies.^12^ We have chosen the descriptor ‘White Paper’ as this seems aligned with our intention of offering expert guidance on differing facets of a high-value clinical topic area.

To ensure the rigour and transparency of our methodology, we incorporated the ACCORD (ACcurate COnsensus Reporting Document)^13,14^ recommendations when defining the methodology for producing White Papers. The ACCORD guideline was developed to promote transparency and uniformity in reporting consensus methods by unpacking the elements in consensus processes and explaining why and how to describe them. This framework supports the development of robust and reliable recommendations by emphasising the importance of transparent reporting, rigorous methodology, and stakeholder engagement.

**Figure 1.**
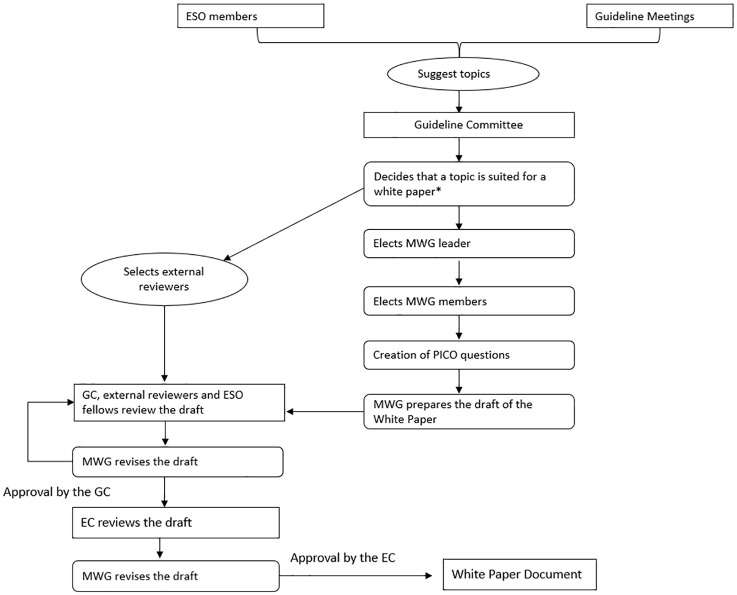
Flow chart of the process of the ESO White Papers. ESO: European Stroke Organisation; GC: Guidelines Committee; MWG: Module Working Group; EC: Executive Committee. *Possibility to choose a White Paper format instead of a standard guideline if it becomes clear that there is insufficient evidence for evidence-based recommendations after preliminary literature scoping.

Initial methods for a White Paper, including the choice of chairs for the guideline and the formulation of the module working group, will be common to the generic SOP for ESO guidelines. PICO (Patient, Intervention, Comparison, Outcome) questions are used to frame and answer clinical and healthcare-related questions by identifying the specific patient population, the intervention under consideration, the comparison or control group, and the outcomes of interest. The creation of PICO questions is essential for guiding the systematic approach to guideline development, and the preliminary search is conducted based on these PICO questions (Figure 1).

If it has not already been decided at the initiation of the work whether a topic would be suited to the White Paper format, it may become clear after literature searching that insufficient materials are available to support evidence-based recommendations for the chosen PICO questions. The final choice of whether to pursue a standard ESO Guideline or a White Paper will be made by the MWG chairs in discussion with the Guideline Board. GRADE (Grading of Recommendations, Assessment, Development, and Evaluations) is a systematic approach to rating the certainty of evidence in systematic reviews and other evidence syntheses.^15^ If the final guidance will likely have less than one-fifth of PICOs supported by an evidence-based GRADE recommendation, then the White Paper format may be preferable. We believe the suggested approach is a reasonable trade-off between prioritising evidence-based recommendations and accepting that sometimes these are not possible.

In making choices around commissioning or supporting White Papers, it should be noted that the primary output of the ESO Guideline group should remain traditional Guidelines that follow the standard SOP.

## Controversies around consensus

The epistemology of producing consensus statements is rooted in systematically collecting and synthesising stakeholder knowledge, experience, and expertise to reach agreed clinical recommendations. This approach recognises the value of expert opinion, particularly in areas where evidence is lacking or inconclusive. In producing our White Papers, this is the approach we wish to follow; recognising the limitations of traditional evidence grading systems, our methodology seeks to fill an essential gap in clinical guidance.

We acknowledge that consensus-based guidance does not replace recommendations based on a robust synthesis of published evidence. Indeed, there are many examples in stroke care where initial ‘expert’ views were subsequently disproven by randomised controlled trials – for example, historical approaches to venous thrombosis prevention or very early mobilisation following disabling stroke.^16,17^ Careful consideration is needed before embarking on a consensus guidance piece. However, it is also important to recognise that clinicians are required to make decisions daily, often in areas where robust evidence is lacking or where existing evidence is inconclusive or not directly applicable. In these scenarios, clinicians already rely on a combination of the best available evidence, clinical experience, and expert guidance to inform their decisions. The aim of a White Paper is to offer a synthesis of all of these sources while always acknowledging the need for better quality evidence and recognising that an expert consensus statement does not remove the equipoise required for a trial. The use of consensus guidance remains controversial, but what is not controversial is that if a consensus is to be developed, then the process should be standardised, transparent and follow recognised international best practices.

## Evidence synthesis for White Papers

Although the intention of a White Paper is not to produce an evidence-based recommendation, there is still a need for the expert consensus to be based on the available evidence. Thus, relevant papers will be collated in the initial literature search. In addition to usual evidence sources, when a decision is made to pursue a White Paper, the search will be expanded to include high-quality reviews on the topic and any appropriate consensus statements published by other professional societies. These will be shared with the MWG to inform the creation of draft guidance statements.

## Methods for producing White Paper consensus statements

A common criticism of consensus materials is a lack of rigour and transparency around the consensus-setting process.^5,18^ Here, we will detail a standardised approach to consensus development. We will allow MWGs a degree of flexibility, but any deviation from the methods described here will need to be described and justified. Ultimately the final document must include a description of the consensus statements’ development with sufficient detail to allow complete independent process replication.

We recognise that the consensus process outlined here differs from that used in the standard ESO Guideline SOP.^9^ We believe the increased level of rigour required for a White Paper consensus versus a Guideline opinion statement is appropriate as the White Paper is based solely on expert opinion.

The formulation of expert consensus statements for ESO White Papers will follow a two-stage process. The MWG will develop a series of draft statements in the first stage. These will be based on the PICO formatted clinical questions agreed upon by the MWG. In an open meeting, the committee will review the evidence from literature searching, discuss their own current practice, and describe what they believe to be the best clinical practice. The MWG will then draft a list of preliminary statements relevant to each PICO question. An emphasis should be placed on practical and clinically relevant advice. In the second stage these draft recommendations will be assessed and revised using a modified Delphi process described below.

### The modified Delphi process

ESO committees are formulated and run with an ethos that all members have equal status. However, evidence suggests that, in many situations, some group members may not feel confident to give their views in an open forum. Equally, individuals may be unwilling to retract long-held views or voice opinions contradicting current practice. In contrast, other, more confident group members may, consciously or inadvertently, dominate the discussion. This can create a situation where recommendations are biased towards the opinions of select group members.^19^ For these reasons, our approach to achieving consensus will use a modification of the Delphi technique with a secret ballot conducted at each round of voting.

The Delphi panel will rate each draft statement based on what they believe represents ‘best clinical practice’. A 9-point Likert scale will be used for rating, with levels from 1 ‘strongly disagree’, through 5 ‘neither agree nor disagree’, to 9 ‘strongly agree’. All members will be encouraged to vote, but an option to abstain from voting due to insufficient knowledge will be offered. In addition to the Likert rating, respondents will be given the opportunity for free text comments on the wording of statements and in the first round of voting, will have the option to propose new revised guidance statements.

After each round of voting, the central ESO administrative team will collate responses, calculate the overall percentage agreement and summarise the written feedback. Ratings will be categorised, where scores of 1–3 will be classed as ‘disagree’, 4–6 ‘equipoise’ and 7–9 ‘agree’. A threshold of 80% agreement is often used to indicate group agreement, whereas less than 50% agreement typically suggests that the statement should be excluded from further voting rounds, unless written feedback provides modifications that could enhance consensus.^19^ While these thresholds are recommended, the MWG may pre-define alternative thresholds for consensus and rejection in specific cases prior to voting.

Each statement will have a minimum of two rounds of voting, even if a ‘consensus’ is reached on the first round. This allows for iteration between rounds and for panellists to reconsider their voting in light of others’ anonymous votes and comments. Where the wording of a statement has been substantially altered following feedback, this will be considered a new item. Items achieving agreement in the first round, but not the second round, will be labelled as ‘unstable agreement’ and will require a minimum further round of revision. All voting members will receive a summary of any wording changes that resulted from the feedback.

**Figure 2.**
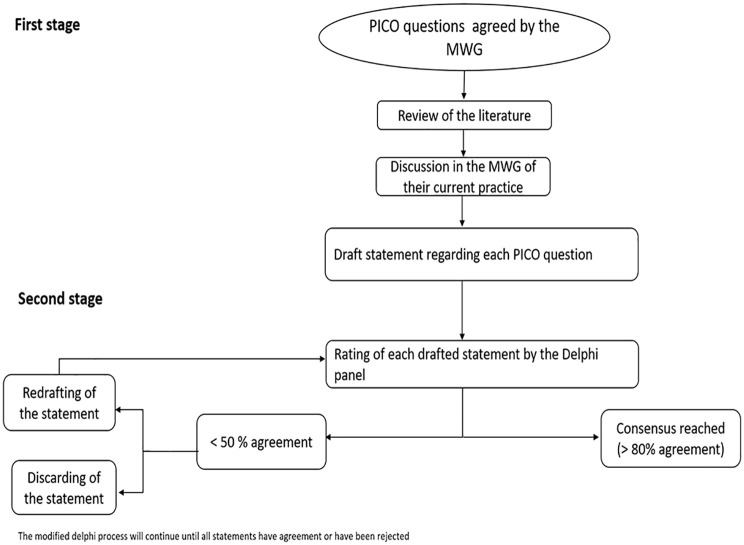
Flow chart of the modified Delphi process. PICO: Patient (or Population), Intervention, Comparison (or Control), and Outcome – is a framework used to formulate clinical questions and guide the systematic search for evidence in the development of guidelines and recommendations; MWG: module working group.

After the voting, all remaining statements will be re-drafted, based on feedback and then resubmitted for a further voting round. The process will continue until all statements have agreement or have been rejected. There will be no maximum number of rounds of voting, but MWG Chairs can choose to end voting if they feel no consensus will be reached. Based on experience with other consensus exercises, the anticipation is that three rounds of voting should be sufficient in most cases. At a final open module working group meeting, the agreed statements will be discussed, and minor modifications can be made to the wording (Figure 2).

Following this meeting, or as part of the meeting, there will be a ratification stage. The entire MWG will engage in the process, where all members review and approve the final statements, confirming the clinical relevance, feasibility, and consistency of the recommendations. The ratification process will also verify that the statements adequately reflect the consensus reached during the discussions.

The agreed text will be shared with the ESO Executive as a final step. This step ensures that the guidance aligns with the organisation’s overarching strategy, mission, and values. Major changes to the guidance are not expected at this stage, and the process of frequent internal ESO review is intended to highlight problematic areas early in the development of the guidance.

## Formulating the modified Delphi group

For standard ESO Guidelines, the MWG consists of up to 10 voting members. The core MWG for a White Paper should be of equivalent size. However, the best practice in Delphi consensus is that the voting panel should be large enough to allow for a range of viewpoints. Thus, a group larger than the MWG will assist with the voting. This group will be referred to as the Delphi panel.

The MWG will have the opportunity to vote on draft statements, but additional Delphi panel members will be invited to participate. These specific Delphi panellists will only assist with the voting and will have no role in other aspects of drafting the White Paper. Delphi panel size has no generally agreed standards, and a range of 20–30 participants is common.^19^ For ESO White Papers, a minimum of 20 active voters will be required.

We propose a multimodal recruitment process to ensure diversity in the Delphi panel. We offer guidance on composition and recruitment whilst recognising the need for flexibility. The final decisions will be made by the MWG Chairs and then agreed with the ESO Guideline Board and Executive Committee. MWG Chairs will suggest members for the Delphi group. MWG members will also be encouraged to nominate potential Delphi group participants. The ESO website will host an open call for Delphi group volunteers specific to a topic, and if needed, the ESO Guideline Board will use their contacts to ensure sufficient (minimum 20) voting numbers are achieved. If there are more applicants than the maximum group number preferred by the MWG Chairs, then the Chairs will have the final choice of participants. A minimum of 10 years of clinical experience will be a requirement for all clinical participants, and MWG Chairs may set other topic-specific criteria. Delphi panellists will not be reimbursed for their time. It is suggested that aggregated data on demographics, speciality, geographical location and career stage of the Delphi panellists is included in the supplement to ensure transparency and highlight the diversity of the group.

We will collate information on speciality, geographic location, career stage, and demographics for both MWG members and Delphi panellists. Although no formal targets will be established, MWG Chairs should endeavour to ensure a broad spread of representation.^20^

Inclusion of stroke survivors or others with lived experience is an increasingly important part of guideline production. We would strongly encourage groups developing their panel to consider including experts with lived experience in various aspects of the process, for example, choosing outcomes of greatest priority, commenting on the face validity of statements and assisting with plain language summaries. In keeping with guidance from other guideline producers, we would not expect the stroke survivors to vote on clinical recommendations.^21^

Where useful to the groups, ESO can signpost to relevant societies and resources that may help with this aspect. While we support including the voice of lived experience and expect this to be considered in every ESO guideline, we do not mandate this inclusion. The choice of stroke survivor representative, as with all other aspects of formulating the group and Delphi panel, is at the discretion of the MWG Chairs. All decisions on membership will be agreed with the ESO Guideline Board and Executive Committee.

## Reporting format of White Papers

Reporting will follow best practices using the ACCORD (ACcurate COnsensus Reporting Document) reporting guidance.^13^

**Title:** The Title will take the format: European Stroke Organisation (ESO) White Paper on <insert topic> with a subtitle of ‘Expert Consensus-Based Clinical Guidance’.

**Introduction:** The Introduction will define the topic area, including the need for guidance. This section should also justify why a formal consensus approach was taken and define the scope of the guidance offered.

**Methods:** Many aspects of the method reporting will be similar to the traditional ESO Guideline SOP. However, there are certain mandatory aspects of method reporting specific to the White Paper format:

- Criteria for selecting Delphi panellists and recruitment process.
- The choice and justification of thresholds that define group consensus (and rejection)
- All the materials shared with the group before formulating the question(s) and voting on consensus statements should be described (e.g. consensus statements from other guidelines, topic reviews, and any available evidence).
- The composition of the Delphi voting group.
- Any deviation or modification of the standard process outlined here should be described and justified.

**Results:** To show that White Papers are distinct from standard clinical practice guidelines, the formatting of results will not use GRADE style templates. To allow for comprehensive and transparent descriptions of the iterative process that informed a consensus statement, the Results section should include a flow chart illustrating the stages of the process, including initial MWG meetings to plan statements, rounds of voting, and internal/external review of the White Paper statements.

Results for each voting round should be reported separately in the main document or appendix. This includes figures showing the average group response, changes in text between rounds, and any free text comments or feedback.

Consensus does not necessarily imply the ‘correct’ answer and lack of consensus and stable disagreement can equally provide insights and highlight important differences in perspectives concerning the topic. Those statements where no agreement could be reached will be included as an appendix, as these may represent areas with the greatest need for new evidence production.

**Figure 3.**
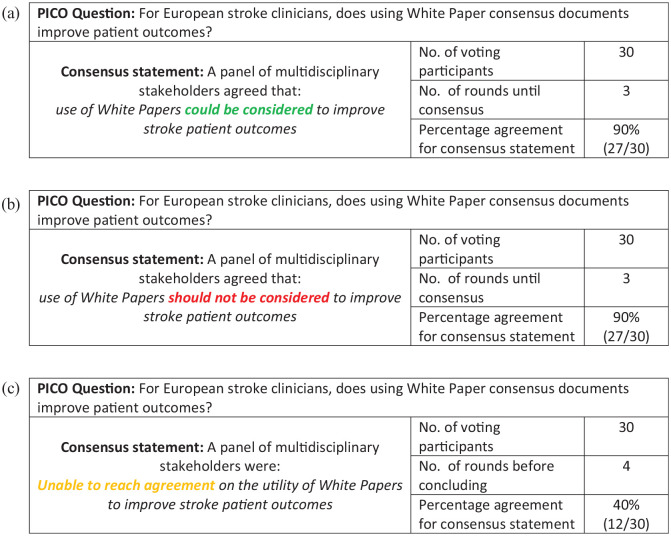
Examples of presentation of differing results from the Delphi process : (a) An example of consensus reached for a recommendation in favour of an intervention, (b) an example of consensus reached for a recommendation not in favour of an intervention, (c) is an example where consensus could not be reached.

The presentation of Results for statements with varying agreement is outlined in Figure 3(a)–(c).

**Discussion:** The Discussion section should include critical reflection on the strengths and limitations of the consensus guidance.

## Authorship on White Papers

The Chairs, MWG and methodologists will all be offered authorship on the White Paper, subject to sufficient participation in producing the guidance as defined by the International Committee of Medical Journal Editors.^22^ Delphi participants will be acknowledged in any resulting materials but will not automatically qualify for authorship. A list of the names and affiliations of all who participated in the Delphi voting group will form a supplement to the White Paper.

## Updates to White Papers

ESO Guidelines have an agreed SOP for regular updating of clinical practice guidelines. Due to the nature of the topic areas and the unpredictable availability of evidence, mandatory updating at regular intervals will not be expected for White Papers. However, where new evidence becomes available, a consensus-based statement should be revised to the format of an evidence-based recommendation. In this scenario, the White Paper will be retired and replaced by a clinical practice guideline, following the usual process for a guideline update.

## Disclaimer

All White Papers and other outputs based only on consensus will have the following disclaimer:

*In the absence of robust evidence, the clinical guidance offered here is based on group consensus. ESO White Papers strive to offer practical clinical suggestions, but these are not evidence-based guideline recommendations and should not be considered mandatory. Clinicians should choose how to use the suggestions contained in a White Paper in the context of the individual patient. ESO accept no liability if the actions suggested are associated with harm or unintended consequences.*
